# Systemic Overexpression of GDF5 in Adipocytes but Not Hepatocytes Alleviates High-Fat Diet-Induced Nonalcoholic Fatty Liver in Mice

**DOI:** 10.1155/2021/8894685

**Published:** 2021-01-15

**Authors:** Yan Yang, Wenting Zhang, Xiaohui Wu, Jing Wu, Chengjun Sun, Feihong Luo, Zhou Pei

**Affiliations:** ^1^Department of Endocrinology and Inherited Metabolic Diseases, Children's Hospital of Fudan University, Shanghai 201102, China; ^2^State Key Laboratory of Genetic Engineering and National Center for International Research of Development and Disease, Institute of Developmental Biology and Molecular Medicine, Collaborative Innovation Center of Genetics and Development, School of Life Sciences, Fudan University, Shanghai 200433, China

## Abstract

**Objective:**

Our recent study demonstrated that growth differentiation factor 5 (GDF5) could promote white adipose tissue thermogenesis and alleviate high-fat diet- (HFD-) induced obesity in fatty acid-binding protein 4- (Fabp4-) GDF5 transgenic mice (TG). Here, we further investigated the effects of systemic overexpression of the GDF5 gene in adipocytes HFD-induced nonalcoholic fatty liver disease (NAFLD).

**Methods:**

Fabp4-GDF5 TG mice were administered an HFD feeding. NAFLD-related indicators associated with lipid metabolism and inflammation were measured. A GDF5 lentiviral vector was constructed, and the LO2 NAFLD cell model was induced by FFA solution (oleic acid and palmitic acid). The alterations in liver function, liver lipid metabolism, and related inflammatory indicators were analyzed.

**Results:**

The liver weight was significantly reduced in the TG group, which was in accordance with the significantly downregulated expression of TNF*α*, MCP1, Aim2, and SREBP-1c and significantly upregulated expression of CPT-1*α* and ACOX2 in TG mouse livers. Compared to that of cells in the FAA-free control group, LO2 cells with in situ overexpression of GDF5 developed lipid droplets after FFA treatment; the levels of triglycerides, alanine aminotransferase (ALT), and aspartate aminotransferase (AST) were significantly increased in both the GDF5 lentivirus and control lentivirus groups compared with those of the FAA-free group. Additionally, the levels of FAS, SREBP-1, CPT-1*α*, and inflammation-associated genes, such as ASC and NLRC4, were unaltered despite GDF5 treatment.

**Conclusion:**

Systemic overexpression of GDF5 in adipose tissue in vivo significantly reduced HFD-induced NAFLD liver damage in mice. The overexpression of GDF5 in hepatocytes failed to improve lipid accumulation and inflammation-related reactions induced by mixed fatty acids, suggesting that the protective effect of GDF5 in NAFLD was mainly due to the reduction in adipose tissue and improvements in metabolism. Hence, our study suggests that the management of NAFLD should be targeted to reduce the overall amount of body fat and improve metabolic status before the progression to nonalcoholic steatohepatitis occurs.

## 1. Introduction

Nonalcoholic fatty liver disease (NAFLD) is a metabolic disease, the incidence of which has rapidly increased in recent years [1]. NAFLD is caused by fat accumulation in hepatocytes, and when NAFLD is insufficiently controlled, it may progress to nonalcoholic steatohepatitis (NASH) [2]. NASH is a kind of progressive fatty liver disease that may worsen with time and eventually lead to serious complications such as cirrhosis, hepatocellular carcinoma (HCC), and liver failure [3]. NAFLD is the most common cause of chronic liver disease in Western developed countries, with an incidence of approximately 30% [4]; the incidence of NAFLD in China is expected to double over the next 10 years [5], exacerbating the occurrence and development of type 2 diabetes and cardiovascular diseases [6]. The etiology of NAFLD is complicated [7], with several therapeutic strategies currently available. However, most treatment modalities have demonstrated minimal effects, and long-term treatment is associated with safety issues [8]. Hence, it is essential to further elucidate the pathogenesis of NAFLD to determine potential prevention and treatment strategies. NAFLD pathogenesis is complex and is not completely understood. The former “double strike” theory did not completely explain the disease and has been replaced by “multiple strike” theory [9]. Current studies have confirmed that NAFLD is mainly associated with insulin resistance, obesity, type 2 diabetes, intestinal flora, and other metabolic disorders [10].

In our previous study, we found that the weight and body mass index (BMI) of adolescents are related to single nucleotide polymorphisms in growth differentiation factor 5 (GDF5) [11]. Later, Hinois et al. showed that brown adipogenesis and energy homeostasis were both positively regulated by the GDF5-Smad signaling pathway in adipose tissues [12]. Our recent study using fatty acid-binding protein 4- (Fabp4-) GDF5 transgenic (TG) C57BL/6J mice showed that TG mice developed a relatively lean phenotype on a high-fat diet (HFD), did not develop metabolic syndrome, and demonstrated increased insulin sensitivity [13]. Here, we further investigated whether overexpression of GDF5 in adipose tissues could affect NAFLD development in mice under HFD conditions and examined the direct effects of GDF5 on NAFLD in an in vitro cellular model.

## 2. Materials and Methods

### 2.1. Fabp4-GDF5 TG Mouse Generation and Treatment

TG mice (GDF5 cDNA (Mouse) (NM_008109.1, Guangzhou Fulen Company, China) were generated as previously described and were maintained by continuous backcrossing, with wild-type (WT) mice serving as controls [13]. All animals were housed in animal facilities under a 12 h light/dark cycle with access to standard chow and water. Mice were fed a standard irradiated rodent chow diet (Slacom, China) or a 60% kcal HFD (Research Diets, New Brunswick, NJ) for 10 weeks starting from 6 weeks of age. Blood was collected after overnight fasting through submandibular venipuncture and was stored at −20°C. Following euthanasia, the liver was extracted and weighed. All animal experiments were approved by the Animal Care and Use Committee of the Children's Hospital of Fudan University.

### 2.2. Histopathology

The mouse liver tissues were fixed overnight in 4% paraformaldehyde at 4°C, embedded in paraffin, sliced into 5 *μ*m sections, stained with HE according to protocols, examined microscopically for histopathological changes, and photographed with a transmission electron microscope according to routine procedures. Fresh liver tissues were embedded in an optimal cutting temperature (OCT) compound (Sakura, Japan) and cryosectioned. The sections were fixed in 4% paraformaldehyde in PBS and stained with 0.5% Oil Red O according to standard procedures.

### 2.3. GDF5-Overexpressing Lentivirus Packaging

The pCDH-CMV-MCS-EF1-Puro plasmid was labeled with 2 × FLAG at the C-end, and the PCR primers of pCDH-C-2 × flag were as follows:

F: TAGAGCTAGCGAATTATGAGACTCCCCAAACT and I: CGTACGCGTGCGGCCCCTGCAGCCACACGATCGTGTGGCTGCAGGGGCCGCACGCGTACG. GDF5 cDNA (NM_001319138.1, Genesent, Hubei, China) was used as the template, and the GDF5 coding sequence was obtained through PCR. The pCDH-CMV-MCS-EF1-Puro vector was digested by XbalI and EcoRI, and the GDF5 coding sequence was ligated to the pCDH-CMV-MCS-EF1-Puro vector. After sequencing identification, pCDH-CMV-MCS-EF1-Puro-GDF5 was used to transfect 293T packaging cells with Lipofectamine 2000. The supernatant was collected after 48 h and filtered through a 0.45 *μ*m filtrate membrane. The supernatant was used to infect LO2 cells for 48 h. After 48 h, 2 *μ*g/ml puromycin (InvivoGen, USA) was added to screen the stably GDF5-overexpressing cell lines [14].

### 2.4. Cell Culture

LO2 cells (donated by Professor Zhao Yan, Laboratory of Liver Cancer Research Institute, Zhongshan Hospital) were cultured in high-glucose Dulbecco's modified Eagle's medium (DMEM) culture medium (Gibco, USA) containing 10% FBS (Gibco, USA). Cells were maintained in a 5% CO_2_ atmosphere with 90% relative humidity at 37°C.

### 2.5. Biochemical Analysis

Triglycerides, aspartate aminotransferase (AST), and alanine aminotransferase (ALT) were analyzed using commercial kits according to the manufacturer's protocols (Nanjing Jiancheng Biological Products Research Institute, China).

### 2.6. NAFLD Model

LO2 cells were seeded in 96-well plates at a density of 10^5^ cells per well, and each well was treated with 0, 0.25, 0.5, 1.0, and 2.0 mmol/L FFA solution (sodium oleate and sodium palmitate (OA:PA = 2 : 1, Sigma, USA)). The treated cells were incubated at 37°C in a 5% CO_2_ atmosphere for 24 h to induce fatty degeneration [15].

### 2.7. Oil Red O Staining

LO2 cells were washed twice with phosphate-buffered saline (PBS), fixed with 4% neutral formaldehyde for 30 min, stained with Oil Red O (Sigma, USA) solution for 15 min, and then, washed twice with distilled water. Images were obtained by a microscope, and the lipid droplets were quantified by the absorbance at 490 nm using an enzyme-labeled meter (BioTek, USA).

### 2.8. Quantitative Real-Time PCR

Total RNA was isolated using a TRIzol kit (Invitrogen, USA). Reverse transcription was performed with a PrimeScript RT master mix and SYBR premix Ex Taq II (Tli RNaseH Plus) (Takara, Japan) according to the manufacturer's recommendations. qPCR was performed using SYBR Green Supermix (Takara) on a Roche thermocycler. Relative expression was analyzed using the **△△**CT method, and the levels were normalized to the housekeeping gene *β*-actin. The primer sequences used in this study are listed in Tables 1 and 2.

### 2.9. Western Blotting

Protein was extracted from LO2 cells using RIPA buffer (Thermo, USA) containing PMSF (Weiao, Shanghai, China). The protein concentration was measured by using a BCA kit (Pierce, Thermo, USA). The protein was resolved by SDS-PAGE, transferred to PVDF membranes (Millipore, USA), blocked with 5% nonfat milk for 1-2 h, and incubated with the primary antibodies against FAS (1 : 1000), CPT-1*α* (1 : 500), SREBP-1 (1 : 1000), and PPAR*α* (1 : 1000) (Abcam, USA) overnight at 4°C. *β*-actin (1 : 5000) (Proteintech, USA) was used as an internal control. A Tanon-5200 imaging system (Tanon, China) was used to detect the immunoreactive bands and quantify each sample.

### 2.10. Statistical Analysis

These data were presented as the mean ± standard deviation. The values were analyzed by nonpaired Student's t-tests and one-way ANOVA using GraphPad Prism version 6.0 software (CA, USA). A *P*-value <0.05 was regarded as statistically significant.

## 3. Results

### 3.1. Histological Effects of High-Fat Diet Induction on the Livers of TG Mice

**Figure 1:**
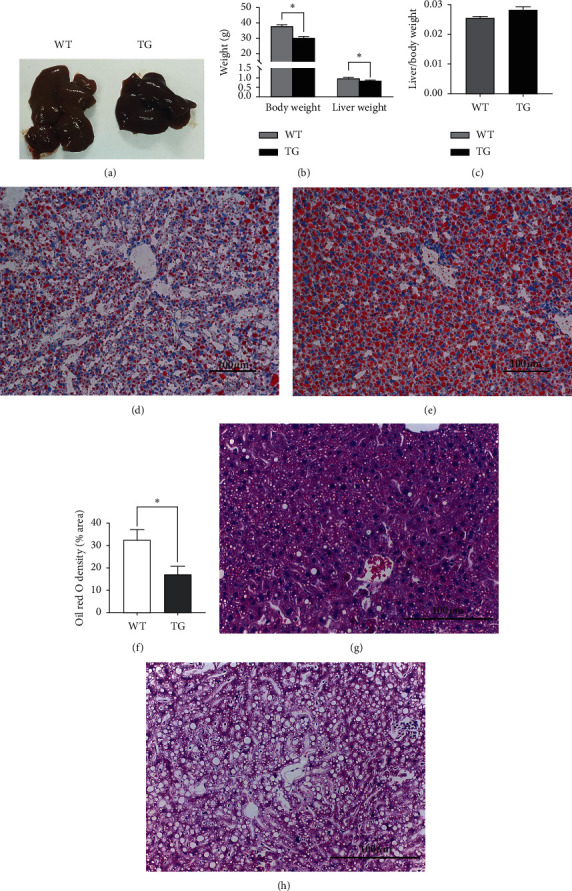
Morphological changes in the livers of GDF5 transgenic (TG) mice after HFD feeding for 10 weeks. (a) Comparison of liver sizes in TG mice and WT mice. (b) Body weights and liver weights of TG and WT mice. (c) Normalization of liver weight to body weight of TG and WT mice. (d) Oil Red O staining of TG mouse livers (scale: 100 *μ*m). (e) Oil Red O staining of WT mouse livers (scale: 100 *μ*m). (f) Quantitative analysis of lipid droplets in hepatocytes. (g) HE staining of TG mouse livers (scale: 100 *μ*m). (h) HE staining of WT mouse livers (scale: 100 *µ*m) (^*∗*^: *P* < 0.05; ^*∗∗*^: *P* < 0.01).

After 10 weeks of HFD feeding, the body sizes of WT mice increased significantly, while TG mice showed a relatively lean phenotype. The liver weights exhibited significant differences between the two groups (Figures 1(a) and 1(b)). When normalized by body weight, the liver weights showed no significant differences between the two groups (Figure 1(c)). However, Oil Red O and HE staining showed that the hepatic cord was well ordered, and hepatocyte morphology looked nearly normal in TG mice (Figures 1(d) and 1(g)). Quantitative analysis of lipid droplets in hepatocytes showed improved liver steatosis in TG mice compared with WT mice (Figure 1(f)). In WT mice, disordered hepatic cords, vacuolar degeneration, and cytoplasmic loosening were observed in hepatocytes (Figures 1(e) and 1(h)).

### 3.2. Changes in Liver Biochemical Markers and the Effect of GDF5 on Lipid Metabolism and Inflammation-Related Genes in TG Mice

**Figure 2:**
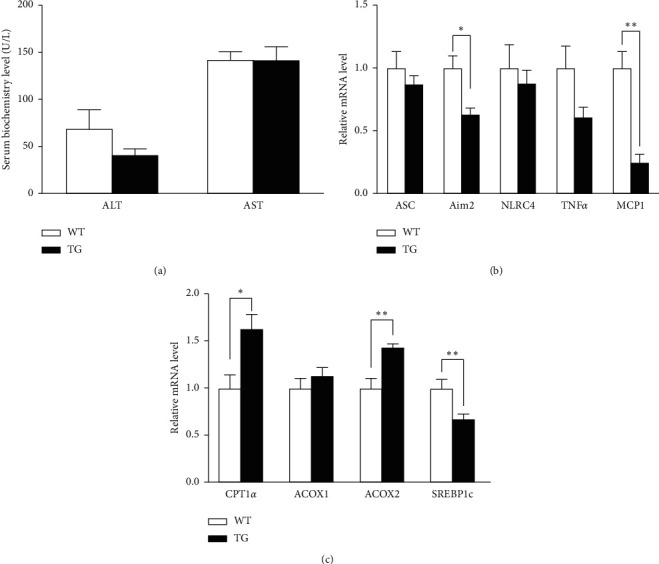
Biochemical markers of fatty liver and liver inflammation in GDF5 transgenic mice. (a) Fasting plasma ALT and AST levels after HFD feeding for 10 weeks. (b) The expression of inflammatory markers in the livers of WT and TG mice. (c) The expression of key enzymes associated with fatty acid oxidation in the WT and TG groups (^*∗*^: *P* < 0.05; ^*∗∗*^: *P* < 0.01).

A slight decrease in ALT expression was detected in TG mice, while no significant difference was found between the two groups (Figure 2(a)). The mRNA levels of TNF*α*, MCP1, Aim2, and SREBP-1c were significantly decreased in the TG group (Figures 2(b) and 2(c)). The expression levels of CPT-1*α* and Acox2 were higher in the TG group than in the WT group. There was no significant difference in Acox1 expression between the two groups (Figure 2(c)).

### 3.3. Effects of GDF5 on Biochemical Markers in FFA-Induced LO2 Steatotic Cells

**Figure 3:**
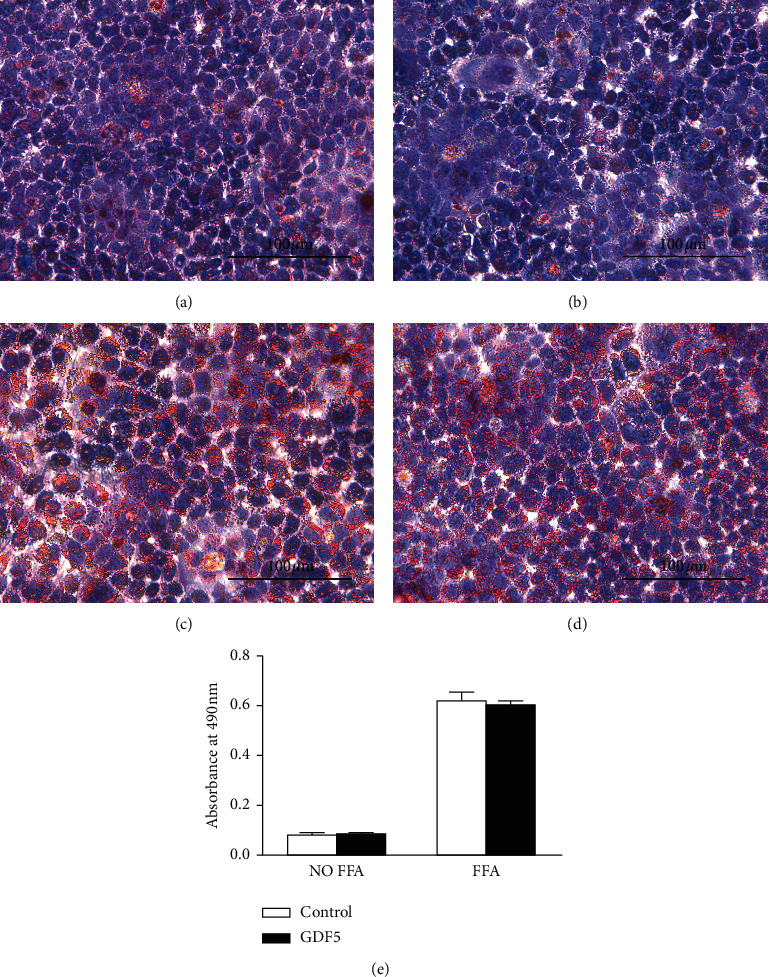
Oil Red O staining of LO2 cell groups with/without FFA treatment (1 mmol/L). (a) LO2 control cells without FFA treatment. (b) Overexpression of GDF5 without FFA treatment. (c) LO2 control cells with FFA treatment. (d) Overexpression of GDF5 with FFA treatment (scale: 100 *μ*m). (e) Quantitative analysis of Oil Red O staining (^*∗*^: *P* < 0.05; ^*∗∗*^: *P* < 0.01).

**Figure 4:**
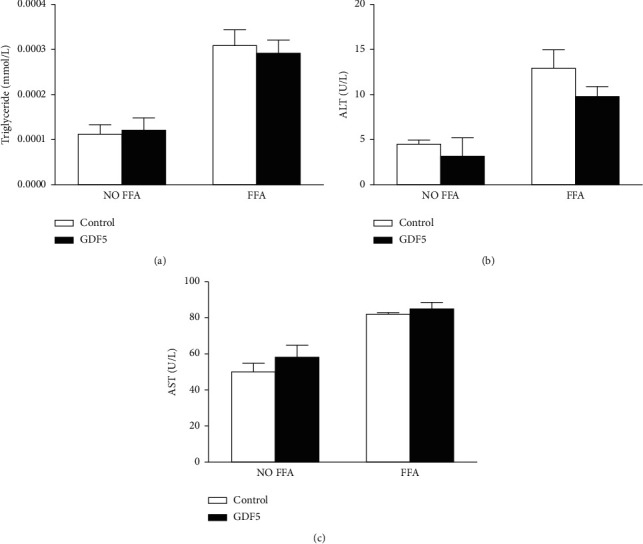
Triglyceride levels and ALT/AST alterations in LO2 cells after FFA induction. (a) The concentration of triglyceride in LO2 cells treated with/without FFA in the GDF5 overexpression and control groups. (b) The concentration of ALT in LO2 cells treated with/without FFA in the GDF5 overexpression and control groups. (c) The concentration of AST in LO2 cells treated with/without FFA in the GDF5 overexpression and control groups (^*∗*^: *P* < 0.05; ^*∗∗*^: *P* < 0.01).

FFA (1 mmol/L) was used to induce fatty degeneration in LO2 cells. After treatment for 24 h, the number of lipid droplets was significantly increased in the FFA-treated groups (Figures 3(a)–3(e)). Consistent with the observed lipid changes, the levels of triglyceride, ALT, and AST were significantly increased compared with those of the FFA-free groups (*P* < 0.05). However, no significant differences in the levels of triglyceride, ALT, and AST were observed in the GDF5-overexpressing group compared with the FFA-treated control group (Figures 4(a)–4(c)).

### 3.4. Effects of GDF5 on Metabolism- and Inflammation-Related Genes in LO2 Cells with Steatosis

**Figure 5:**
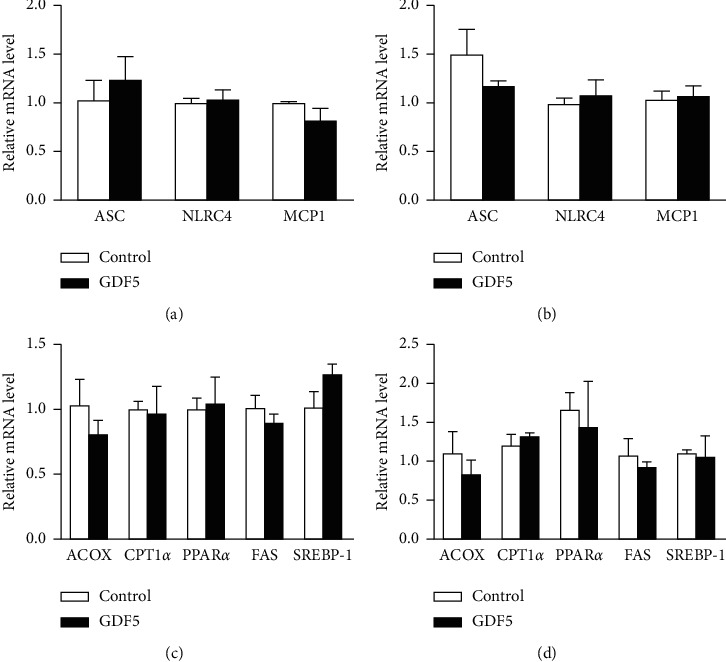
Normalized expression of NAFLD-related genes in LO2 cells. LO2 cells were transfected with a GDF5 overexpression lentivirus. (a) qPCR analysis of inflammation-related genes before FFA treatment. (b) qPCR analysis of inflammation-related genes after FFA treatment. (c) qPCR analysis of lipid metabolism-related genes before and (d) after FFA treatment. (^*∗*^: *P* < 0.05; ^*∗∗*^: *P* < 0.01).

**Figure 6:**
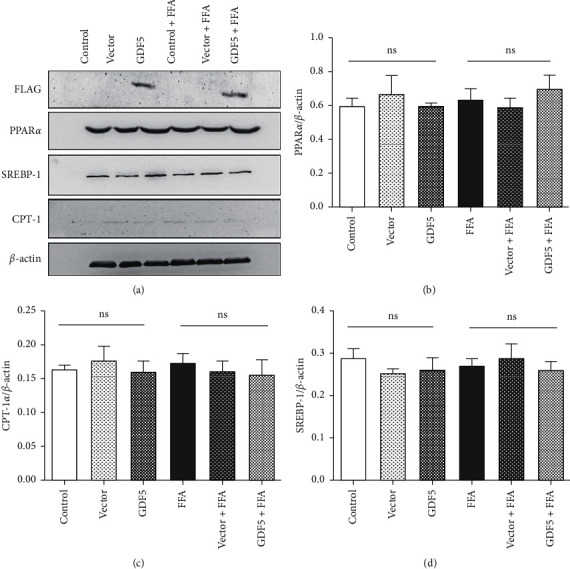
Immunoblot analysis (a) and quantification of NAFLD-related genes in different LO2 cells after FFA induction. Relative expression of PPAR*α*, CPT-1*α*, and SREBP-1; (b) CPT-1*α*: carnitine palmitoyltransferase-1*α*; (c) SREBP-1: sterol regulatory element-binding protein 1; and (d) PPAR*α*: peroxisome proliferator-activated receptor *α*. (^*∗*^: *P* < 0.05; ^*∗∗*^: *P* < 0.01).

We assessed the expression of inflammation-related and NAFLD-related genes in FFA-induced LO2 cells with/without GDF5 overexpression. The expression of ASC, NLRC4, and MCP1 in the FFA treatment group was not significantly different from that in the control group (Figures 5(a) and 5(b)). The expression of the key *β*-oxidation enzymes ACOX and CPT-1*α* increased slightly after FFA treatment. The expression of SREBP-1 and FAS, key genes associated with triglyceride synthesis, exhibited no significant difference between the GDF5 overexpression group and the control group (Figures 5(c) and 5(d)). PPAR*α* was higher than that in the control group after FFA induction, with no significant difference (Figure 5(d)). After 72 h of lentiviral infection, the results showed that the expression of GDF5-flag was significantly increased in the GDF5 and GDF5 + FFA groups compared with the other groups (Figure 6(a)). The protein levels of NAFLD-related genes, such as FAS, CPT-1*α*, PPAR*α*, and SREBP-1, were measured, and the results demonstrated that the levels of these markers in the three FFA-treated groups did not differ significantly from those in the FFA-free group, and no significant differences were observed between the three FFA-treated groups (Figures 6(b)–6(d)).

## 4. Discussion

GDF5 is a member of the TGF-*β* superfamily. Our previous research on GDF5 showed that GDF5 was critical in 3T3-L1 preadipocyte differentiation [16]. Overexpression of GDF5 in adipose tissues resulted in improved insulin sensitivity and metabolic syndrome resistance [13].

In the present study, we used the Fabp4-GDF5 TG mouse model to explore the effects of GDF5 on NAFLD. In Fabp4-GDF5 TG mice, GDF5 was mainly overexpressed in white and brown adipose tissue. In response to HFD feeding, these TG mice exhibited a lean phenotype and increased insulin sensitivity compared with those of WT mice. Tissue-specific gene expression analysis showed that the GDF5 mRNA expression levels in our TG mice were similar to those in WT mice [13]. To our surprise, Oil Red O and HE staining showed that an HFD caused hepatic steatosis in WT mice. The liver weights of TG mice were decreased, as were transaminases and the number of lipid droplets. However, when the weight of the liver was normalized to the body weight, the ratios were not significantly different between the two groups, and the reason for the seemingly inconsistent liver weight and liver weight-to-mouse body weight ratio was the significantly decreased body weights of TG mice during HFD treatment [13].

The detailed mechanism of NAFLD is complex and multifactorial. NAFLD pathogenesis has been explained by the “multiple hit theory,” including insulin resistance, obesity, nutritional factors, gut microbiota, and genetic and epigenetic factors [9]. Lipid accumulation can be induced by increased fatty acid uptake, increased de novo lipogenesis, and decreased fatty acid oxidation, followed by esterification for TG synthesis [17]. Decreased fatty acid oxidation could lead to dysfunctional hepatocyte lipid metabolism and lipid accumulation [18]. CPT-1*α* and ACOX are two key enzymes involved in fatty acid *β*-oxidation [19]. In this study, we detected significantly increased expression of CPT-1*α* and ACOX2 in Fabp4-GDF5 TG mice.

Moreover, NAFLD is closely related to inflammatory responses. Increased FFA levels can cause lipotoxicity and insulin resistance, activating the release of proinflammatory cytokines such as TNF*α*, IL-6, and other factors (such as hypoxia and endogenous endotoxin) in the liver, finally leading to hepatocyte degeneration and necrosis [20].

We observed that inflammatory markers, including TNF*α*, MCP1, Aim2, and SREBP-1c, decreased in HFD-fed TG mice. These results suggested that Fabp4-GDF5 TG mice were protected against HFD-induced NAFLD. Then, we investigated whether the improvement in NAFLD was directly due to systemic GDF5 overexpression in adipocytes. We further examined whether overexpression of GDF5 in LO2 cells could improve metabolism and inflammation in NAFLD. LO2 cells, which are known as normal hepatocytes, are one of the commonly used cell types to establish an NAFLD cell model in vitro [15]. However, no significant protective effects on NAFLD-related marker genes were detected in GDF5-overexpressing LO2 cells after FFA induction.

Obesity is the most important risk factor for NAFLD, and it also increases the risk of advanced disease, including NASH-related cirrhosis and HCC. There is a positive linear correlation between the prevalence of NAFLD and increased BMI [21]. Obesity could also increase liver-specific mortality. It has been observed that liver steatosis was improved with a weight loss of 5% [22]. Additionally, liver steatosis, inflammation of the liver lobule, swelling, and the NAFLD activity score (NAS) are significantly improved with a 9% weight loss [23]. Our findings suggest that the protective effect of GDF5 against NAFLD in vivo can be attributed to the secondary effect of systemic changes in weight loss and metabolic state.

Given that there is no specific drug available for NAFLD treatment in clinical practice [24], it is essential to adopt methods that would eliminate the cause of the disease through lifestyle modifications, as proposed by all guidelines.

Based on our combined in vivo and in vitro results, we suggest that the basic treatment for NAFLD is weight loss and an improvement in overall metabolism, at least, before NAFLD progresses to NASH, and solely targeting the liver is not an optimal therapeutic strategy in NAFLD. However, more studies are needed in this area to augment our understanding of the treatment of NAFLD.

## 5. Conclusions

In conclusion, this study demonstrated that overexpression of GDF5 in adipose tissue led to a lean phenotype and protected against NAFLD. The protective effect of GDF5 against NAFLD was mainly due to the reduction in adipose tissue and improvements in metabolism, rather than direct effects on hepatocytes.

## Figures and Tables

**Table 1:** Primer sequences (human).

Name of genes	Sequences	Size (bp)
TNF *α*	FP: CCCTCACACTCAGATCATCTTCT RP: GCTACGACGTGGGCTACAG	61

MCP1	FP: TTAAAAACCTGGATCGGAACCAA RP: GCATTAGCTTCAGATTTACGGGT	21

ASC	FP: CTTGTCAGGGGATGAACTCAAAA RP: GCCATACGACTCCAGATAGTAGC	154

Aim2	FP: GTCACCAGTTCCTCAGTTGTG RP: CACCTCCATTGTCCCTGTTTTAT	213

CPT1*α*	FP:CTCCGCCTGAGCCATGAAG RP: CACCAGTGATGATGCCATTCT	100

ACOX-1	FP: TAACTTCCTCACTCGAAGCCA RP: AGTTCCATGACCCATCTCTGTC	283

ACOX-2	FP: ACGGTCCTGAACGCATTTATG RP: TTGGCCCCATTTAGCAATCTG	125

SREBP-1c	FP: GCAGCCACCATCTAGCCTG RP: CAGCAGTGAGTCTGCCTTGAT	199

NLRC4	FP: GAAACACTGTACGATCAGCTCC RP: CATGTTCTTGAAGCGATGGTTTT	183

*β*-actin	FP: GGAGATTACTGCCCTGGCTCCTA RP: GACTCATCGTACTCCTGCTTGCTG	150

**Table 2:** Primer sequences (human).

Name of genes	Sequences	Size (bp)
SREBP-1	FP: ACCGTTTCTTCGTGGATGG RP: ACACAGTTCAGTGCTCGCTC	145

FAS	FP: GACAGAGCAACTACGGCTT RP: CTCATCGTCTCCACCAAA	133

CPT-1*α*	FP: ATCAATCGGACTCTGGAAACGG RP: TCAGGGAGTAGCGCATGGT	121

ACOX	FP: TGCTCAGAAAGAGAAATGGC RP: TGGGTTTCAGGGTCATACG	132

MCP1	FP: CGCCTCCAGCATGAAAGTCT RP: GGAGGCCATTCAGGGTCAG	66

NLRC4	FP: TCAGAAGGAGACTTGGACGAT RP: GGAGGCCATTCAGGGTCAG	193

PPAR*α*	FP: GCTATCATTACGGAGTCCACG RP: TCGCACTTGTCATACACCAG	88

ASC	FP: GACCTCACCGACAAGCTG RP: CCGGTGCTGGTCTATAAAGTG	150

*β*-Actin	FP: GGCACCACACCTTCTACAAT RP: AACATGATCTGGGTCATCTTCTC	115

## Data Availability

The data used to support the findings of this study are available from the corresponding author upon request.
